# How Well Are Malaria Maps Used to Design and Finance Malaria Control in Africa?

**DOI:** 10.1371/journal.pone.0053198

**Published:** 2013-01-11

**Authors:** Judy A. Omumbo, Abdisalan M. Noor, Ibrahima S. Fall, Robert W. Snow

**Affiliations:** 1 Malaria Public Health Department, Kenya Medical Research Institute/Oxford University/Wellcome Trust Research Programme, Nairobi, Kenya; 2 Centre for Tropical Medicine, Nuffield Department of Clinical Medicine, University of Oxford, Oxford, United Kingdom; 3 Disease Prevention and Control Cluster, World Health Organization, Regional Office for Africa, Brazzaville, Republic of Congo; Tulane University School of Public Health and Tropical Medicine, United States of America

## Abstract

**Introduction:**

Rational decision making on malaria control depends on an understanding of the epidemiological risks and control measures. National Malaria Control Programmes across Africa have access to a range of state-of-the-art malaria risk mapping products that might serve their decision-making needs. The use of cartography in planning malaria control has never been methodically reviewed.

**Materials and Methods:**

An audit of the risk maps used by NMCPs in 47 malaria endemic countries in Africa was undertaken by examining the most recent national malaria strategies, monitoring and evaluation plans, malaria programme reviews and applications submitted to the Global Fund. The types of maps presented and how they have been used to define priorities for investment and control was investigated.

**Results:**

91% of endemic countries in Africa have defined malaria risk at sub-national levels using at least one risk map. The range of risk maps varies from maps based on suitability of climate for transmission; predicted malaria seasons and temperature/altitude limitations, to representations of clinical data and modelled parasite prevalence. The choice of maps is influenced by the source of the information. Maps developed using national data through in-country research partnerships have greater utility than more readily accessible web-based options developed without inputs from national control programmes. Although almost all countries have stratification maps, only a few use them to guide decisions on the selection of interventions allocation of resources for malaria control.

**Conclusion:**

The way information on the epidemiology of malaria is presented and used needs to be addressed to ensure evidence-based added value in planning control. The science on modelled impact of interventions must be integrated into new mapping products to allow a translation of risk into rational decision making for malaria control. As overseas and domestic funding diminishes, strategic planning will be necessary to guide appropriate financing for malaria control.

## Introduction

Maps provide a powerful visual tool to identify areas where targeted strategies and resources are most likely to have the greatest impact. The 1960s saw the most informed malaria mapping exercise of the 20^th^ century undertaken by two Soviet malariologists involving a major synthesis of historical records of malaria, reported prevalence of sickle cell trait, climate information (temperature limitations of transmission), topography (altitude and proximity to water bodies), Ministry of Health (MoH) documents and maps of malaria endemicity based on expert opinion. These data were assembled around ecological “nosoareas” and interpolated globally for malaria at the peak of its assumed historical distribution in 1900 [1]. Within this range categories of risk were used following the hypo to holo-endemic classifications approved at a regional World Health Organization (WHO) malaria conference held in Kampala in 1950, based originally on spleen rates and later adapted to *Plasmodium falciparum* parasite rates in children aged 2–10 years (*Pf*PR_2–10_) [2], and an additional zone “subject to severe epidemics” was also defined. Lysenko and Semashko used the global map in a qualitative sense to consider the status and future of malaria eradication; however they concluded that “(The map) *should reflect the dynamics during the implementation of malaria eradication programmes, or the successful results of campaigns to combat malaria in the countries where they were implemented. This map therefore has to be updated regularly*.”

By the 1970s, following the demise of the Global Malaria Eradication Programme in sub-Saharan Africa, there were few attempts to qualitatively or quantitatively develop malaria risk maps despite Lysenko and Semashko's plea for regular malaria cartography to monitor control and elimination progress. In 1996 a renewed plea for more detailed evidence-based malaria maps to effectively target new vector control methods in Africa led to the formation of a Pan-African collaboration, the Mapping Malaria Risk in Africa collaboration (MARA/ARMA) [3]–[5]. This collaboration published a number of important malaria risk maps based on the suitability of climate for malaria transmission [6] and the average duration (in months) of suitable transmission conditions within a year [7]–[8]. The MARA/ARMA parasite prevalence data archive was also used to generate a modelled prediction of parasite prevalence across West Africa in 2001 [9]. The MARA/ARMA initiative served as a model for the establishment of an initiative called the Malaria Atlas Project (MAP), a collaboration between scientists in Kenya and Oxford, UK in 2005 [10]–[11]. MAP picked up the mantle of data assembly with a focus on the period post-1985 and at a global level. In 2009 a model-based geostatistical approach was implemented to produce an interpolated global map of continuous predicted *Plasmodium falciparum* parasite prevalence (0–100%) for the year 2007 and used to classify malaria risk into areas of *Pf*PR_2–10_<5%, 5–39% and > = 40% and the inclusion of areas unable to support transmission because of aridity, low ambient temperature or where medical intelligence suggested clinical incidence was less that 1 per 10,000 population [12].

Over the last fifteen years there has been a proliferation in the co-availability of national, geo-coded parasite prevalence data and spatially-interpolated climate data derived from ground station observations, remotely sensed satellite surrogates of climate, urbanization and topography. Advances in computing and geostatistical techniques have increased the ability to define the spatial and temporal risks of malaria endemicity using probabilistic approaches at high spatial resolutions and formed the basis of a variety of country-level maps of risk including South Africa [13], Mali [14], Malawi [15], Zambia [16], Somalia [17], Democratic Republic of Congo [18], Kenya [19], Botswana [20], Angola [21], Namibia [22], Uganda [23], Cote D'Ivoire [24], Tanzania [25] and Senegal [26].

During the late 1990s when Africa was in the grips of a spiralling malaria epidemic [27] and coverage of interventions with proven efficacy was poor [28]–[31], the WHO, donors and national governments advocated for wide-scale coverage of a single combination of interventions: insecticide treated nets (ITN), treatment with artemisinin-based combinations (ACTs) and intermittent preventive treatment of malaria in pregnant women (IPTp). These interventions were promoted across all African countries and all communities within each country. As the Global Fund (GF), which currently provides over 75% of malaria Overseas Development Assistance (ODA) [32], becomes increasingly limited in its ability to fund national proposals it will have to rationalize grant approvals more effectively. This will lead to a demand on countries to better define their needs, requirements, targets and domestic commitments to malaria control based on sub-national epidemiological requirements and populations in need.

We examine how malaria risk maps are used by national governments across Africa to design malaria control within their borders through a review of national malaria strategic plans (NMS), malaria programme performance reviews (MPR) and country-led successful funding applications to the GF. The aim of this review is to understand what is used and how, what isn't used and why and what might be improved to foster a more epidemiologically informed approach to malaria control over the next 10–15 years as demands on limited ODA increases.

## Materials and Methods

### Scope and map classifications

This review considers 47 malaria endemic countries in Africa defined as all countries where malaria transmission was reported in 2010 including countries with a stated malaria elimination agenda (Cape Verde and Swaziland). Zanzibar is treated independently of the United Republic of Tanzania as it is governed by a separate MoH and the Government of South Sudan is also recognized following its recent official formation in July 2011. The most recent available NMS for each country, national malaria monitoring and evaluation (M&E) plans, MPRs and applications submitted to the GF were obtained through web-searches and personal communication and examined for the use of maps and whether mapped risk is taken into consideration in planning, decision-making and resource allocation for malaria control. There is a wide variation in the types of maps in use by NMCPs and for simplicity they are classified according to 11 types linked to the data and information on which they are based (Table 1).

**Table 1:** Description of codes for map types used by NMCPs.

0	No map provided in the report.
1	Qualitative definitions of epidemiological conditions used to describe areas as malaria free, endemic, non-endemic, stable, unstable, low-moderate-high endemic or hypo-, meso- hyper- and holo-endemic. These maps are often based on expert opinion and may combine some empirical data in defining of levels of endemicity classes.
2	Maps based solely on eco-climatic determinants of malaria transmission such as temperature, altitude or rainfall. Such maps may describe, for example, the duration (in months) of the transmission season or define altitudinal limits above which transmission may occur or altitudinal ranges within which epidemic outbreaks are frequent.
3	Map of reported number of cases within administrative units (e.g. region or district) based on observed routine health information systems data.
4	Maps of malaria case-incidence rates (usually per 1000 population per year) according to administrative units and developed using nationally reported cases and population census data as a denominator.
5	Maps of reported parasite prevalence according to administrative units usually derived from periodic cross-sectional national household sample surveys.
6	Models of the suitability of climatic conditions (rainfall, temperature and humidity) to support transmission. The theoretical maps, developed by the MARA/ARMA collaboration [7], are based on 60-year climatology (estimates of standard means for the period 1920 to 1980) and threshold values for the climate variables. The first of these is presented as a continuous surface that defines the suitability of transmission ranging from 0 (unsuitable) to 1 (suitable).
7	MARA/ARMA climate suitability map categorized as endemic; marginal/epidemic; malaria-free
8	The MARA/ARMA seasonality model showing the duration of the transmission season (classified as <3 months, 3–6 months or >6 months), start month and end month of malaria transmission season [7].
9	MARA/ARMA parasite prevalence model developed by for West Africa. PfPR data are used as training data in the development of this model [9].
10	1Country-specific tailored models of parasite prevalence based on national prevalence survey data and developed using Bayesian model-based geo-statistical (MBG) methods.
11	Interpolated models of parasite prevalence presented as continuous surfaces or according to classes of unstable vs. stable endemic risk. These are developed on a global scale by the Malaria Atlas Project (MAP) and country-specific maps are available in the public domain [11].

### National Malaria Strategies

The visions, goals, objectives and milestones articulated in a country's NMS allows the government to define evidence-based strategic objectives and interventions and provide a matrix to measure progress. The NMS is therefore a key consensus document that each country uses to guide the implementation and evaluation of malaria control. Following the launch of the Roll Back Malaria (RBM) partnership in 1998, there was a proliferation of new NMS across Africa. These new NMSs, for the most part, followed a set of harmonized objectives around scaled coverage of ITN, chemoprophylaxis for pregnant women, prompt access to efficacious treatment of clinical malaria, improved community awareness of malaria and early detection and actions necessary to contain epidemics as set out during the Abuja declaration [33]. Almost all strategic objectives were set at protecting 60% of at-risk populations, defined largely as children under five and pregnant women, with preventative interventions or effectively treated clinical events with the goal of halving malaria morbidity and mortality by 2010. While important to galvanize countries and donors around a set of regional and global ambitions, very few of the NMS's developed in the early 2000s recognized the diversity of “at-risk” populations within their national boundaries or how this diverse epidemiology necessitates different combinations of interventions and of coverage sub-nationally. This was reflected in initial GF malaria applications from countries in Africa from Round 2 (2002) to Round 6 (2006) that emphasized rapid scale-up of ITN coverage and/or changing national policies to universally support government sector delivery of new ACTs. After 2006, when countries began to re-develop their NMS and refine resource needs, including subsequent applications to the GF, ambitions began to become more sophisticated, better tailored according to national epidemiologies and to include more ambitious goals including the achievement of zero malaria incidence, elimination and removing malaria as a public health threat.

The most recent NMS for each of the 47 NMCPs was identified through web-searches and personal communications. Most countries have developed more than one NMS since 2000 and of these, the most recent launched after 2005 was selected (except in the case São Tomé and Principe 2001–2010). Of the available NMS, 35 (74.5%) are currently operational and cover the period from 2007 through to 2016. The operational period for the NMS of Burundi, Congo, Rwanda, Sudan (North) and Zanzibar will end in 2012. NMS for 12 countries (Benin, Cameroon, Central African Republic (CAR), Côte d'Ivoire, Democratic Republic of Congo (DRC), Djibouti, Gabon, Guinea, Mauritania, Niger, São Tomé & Principe and South Sudan) cover operational periods ending in 2011 and revised strategies for these countries are under development.

NMS were reviewed to identify stated visions, specific objectives and how the NMCP proposes to measure achievement against these objectives. Each NMS was reviewed to ascertain whether maps or descriptions of varied malaria risk were used to select specific malaria strategies and target tailored intervention packages at the sub-national level. We have defined ‘sub-national risk planning’ as the use of geographically defined sub-national spatial units for defining programme objectives, allocating resources, targeting interventions or planning control activities. Such spatial units included national political boundaries or administrative units, health districts or geographically defined areas of malaria risk that encompass a defined denominator population. Where possible, we also examined national malaria M&E plans and MoH annual reports as alternative sources of information on existing or proposed sub-national definition of malaria epidemiological risk. We were able to identify 11 M&E plans linked to concurrent NMS, (Burkina Faso, Ghana, Kenya, Namibia, Nigeria, Rwanda, Somalia, South Africa, Tanzania, Uganda and Zambia) and a MoH Annual Report (2007) for South Africa that provided additional information on the use of risk maps.

### National Malaria Programme Performance Reviews (MPR)

The WHO Africa Regional Office (WHO-AFRO) has supported countries to undertake national consultations and review their progress in malaria control, financing and the achievement of targets since 2009. In September 2011, WHO-AFRO developed a manual to provide guidelines for countries to develop their NMS. The manual includes a recommendation to NMCPs to undertake an MPR [34]. The MPR provides mid and end-term reviews of the achievement of objectives within the NMS and enables programmes to re-adjust activities where necessary in order to keep these aligned with policy targets. It is suggested that the MPR process take a year to complete and include a review of malaria epidemiology and stratification. This includes describing the seasonality of transmission and geographical distribution of malaria burden, parasite prevalence and parasite species [34], [35]. MPRs for 19 (40%) of the countries reviewed were completed by March 2012 and available for this review. Information obtained from the MPRs included whether or not a map was presented, the type of malaria risk map used and whether the risk maps were used in strategic planning. Examples of strategic use of epidemiological risk maps include; documented evidence of the use of maps in defining needs, selecting and targeting interventions, for resource allocation or for outlining monitoring strategies.

### Global Fund applications

Since its inception in January, 2002 the GF has required that every application for malaria funding should be accompanied by a nationally approved strategic plan for malaria control. The GF review process has evolved over the last 10 years with application processes becoming more precise in their demand for information on the epidemiological pattern of malaria risk within a country, although not so distinct in conveying the types of information countries should provide. The guidelines for Round 10 submissions (2010) require that “at-risk” populations are defined at sub-national level and state that: “*For the populations targeted in the proposal, the applicant should provide the most recent population size and epidemiological data relevant to those groups, preferably disaggregated by sex and age, and generated less than five years ago. The applicant may identify other groups as important relying on current epidemiological evidence*” and *“Where key disaggregated data is not available, the proposal should incorporate a plan to acquire this data prior to the Phase 2 review”.*

The most recent successful applications since 2006 (Round 6) from 43 (91%) malaria endemic countries in Africa were downloaded from the GF website [36]. Higher income countries (Botswana and South Africa) have been ineligible for funding since 2002. No successful application was available for either Gabon or Equatorial Guinea during the funding years (2006–2010) reviewed here. The Round 10 proposal labeled as “malaria” for South Sudan available on the GF web site was found to be largely about HIV and the Round 9 application was used instead. Of the 43 applications available for review three were awarded during Round 6 (2006), four in Round 7 (2007), 11 in Round 8 (2008), 12 in Round 9 (2009) and 13 in Round 10 (2010). The applications were examined for use of epidemiological risk, stratifications and/or maps to frame the context of requested support. It is accepted that this review may represent a biased sample given that only successful applications are posted on the GF's website. While it might be true that unsuccessful applications were those that may have inadequately defined their epidemiological risk it is also true that successful applications should have theoretically linked malaria epidemiology to targeted control needs and therefore relevant within this review.

While it might be true that unsuccessful applications were those that may have inadequately defined their epidemiological risk it is also true that successful applications should have theoretically linked malaria epidemiology to targeted control needs and therefore relevant within this review.

## Results

### National Malaria Strategies

Thirty-two (68%) of the countries reviewed presented maps of malaria epidemiology within their NMS. Although risk maps were not provided in the NMS for Kenya and South Africa, alternatives were identified in the national M&E Plan for Kenya, developed simultaneously with the NMS, and a MoH Annual Report (2007) for South Africa developed during the same year as the NMS. Fifteen (32%) of the NMS reviewed did not include a risk stratification map (Benin, Cape Verde, CAR, Chad, Comoros, Republic of Congo, Côte d'Ivoire, Equatorial Guinea, Eritrea, Gabon, Gambia, Guinea, Guinea-Bissau, São Tomé & Principe and Sierra Leone). Fourteen (44%) maps were based on either qualitative epidemiological descriptions or eco-climatic determinants of transmission (map codes 1 and 2; Table 2); 9 (28%) NMS included maps that simply aggregate routine Health Information Systems (HIS) data (confirmed cases and/or incidence) or prevalence survey data according to administrative units (map codes 3–5; Table 2); 4 (12.5%) NMS (Cameroon, South Sudan, Tanzania and Nigeria) used the public domain climate based models of transmission developed by the MARA/ARMA collaboration (map code 8); while 5 (15.5%) NMS used modeled predictions of parasite prevalence based on empirical data including tailored maps developed by national research scientists in collaboration with NMCPs (Ghana, Liberia, Kenya, Namibia and Somalia) (map codes 9 and 10; Table 2). Although 8 of the NMS were launched after the release in 2009 of the first products developed by MAP, they did not include a MAP web-available product.

**Table 2:** Maps used in NMS, GF applications, MPRs; dates of report and evidence of sub-national planning.

Country	NMS#Code - (Date)	Sub-national planning in NMS?	MPR Code - (Date)	GF Code - (Date)	Sub-national planning in GF application?
Angola	**1** - (2008–2013)	N	**-**	**1** - (2010)	N
Benin	**0** - (2006–2010)	N	**-**	**4** - (2007)	N
Botswana	**1** - (2010–2015)	Y (E*)	**3** – (2009)	-	-
Burkina Faso	**4** - (2011–2015)&	Y	**-**	**2** - (2008)	N
Burundi	**1** - (2008–2012)	Y	**0** – (2011)	**0** - (2009)	N
Cameroon	**8** - (2007–2010)	N	**-**	**8** - (2009)	N
CAR	**0** - (2007–2011)	N	**-**	**0** - (2008)	Y
Cape Verde	**0** - (2009–2013)	Y (E*)	**-**	**4** - (2010)	Y (PE)
Chad	**0** - (2009–2013)	N		**7** - (2009)	Y
Comoros	**0** - (2007–2014)	Y (PE**)	**1** – (2011)	**0** - (2008)	Y
Congo (Republic of)	**0** - (2008–2012)	N	**-**	**3** - (2008)	N
Côte d'Ivoire	**0** - (2006–2010)	N	**-**	**4** - (2008)	N
Djibouti	**1** - (2006–2010)	Y	**1** – (2011)	**1** - (2006)	N (E)
DRC	**2** - (2007–2011)	N	**-**	**2**- (2010)	N
Equatorial Guinea	**0** - (2009–2013)	N	-	-	-
Eritrea	**0** - (2010–2014)	Y		**4** - (2009)	N
Ethiopia	**2** - (2010–2015)	Y (PE)	**2** - (2011)	**2** - (2008)	N
Gabon	**0** - (2006–2010)	N	-	-	-
Gambia	**0** - (2008–2015)	N	**-**	**0** - (2009)	N
Ghana	**9** - (2008–2015)&	Y	**-**	**9** - (2008)	Y
Guinea	**0** - (2006–2010)	N		**1** - (2010)	Y
Guinea Bissau	**0** - (2009–2013)	N	**-**	**3** - (2009)	N
Kenya	**10** - (2009–2017)&	Y	**10** - (2009)	**10** - (2010)	Y
Liberia	**9** - (2009–2013)	N	**-**	**5** - (2010)	N
Madagascar	**2** - (2008–2013)	Y	**2** - (2011)	**0** - (2009)	Y (E)
Malawi	**3** - (2011–2015)	N	**10** - (2012)	**0** - (2009)	N
Mali	**2** - (2010–2014)	N	**-**	**2** - (2010)	N
Mauritania	**2** - (2006–2010)	N	**2** - (2011)	**3** - (2006)	Y
Mozambique	**4** - (2011–2016)	Y	**4** - (2010)	**5** - (2009)	N
Namibia	**10** - (2010–2016)&	Y (PE)	**10** - (2010)	**0** - (2006)	N
Niger	**1** - (2006–2010)	Y	**-**	**7** - (2007)	N
Nigeria	**8** - (2009–2013)&	N	**-**	**8** – (2008)	N
Rwanda	**1** - (2008–2012)&	Y (PE)	**5** - (2011)	**1** - (2008)	N
São Tomé & Principe	**0** - (2001–2010)	N	**-**	**3** - (2007)	N (E)
Senegal	**4** - (2011–2015)	Y	**5** - (2011)	**4** - (2010)	Y
Sierra Leone	**0** - (2009–2015)	N	**-**	**9 & 11** - (2010)	N
Somalia	**10** - (2011–2015)&	Y	**-**	**10** - (2010)	Y
South Africa	**4** - (MoH Ann. Rep. 2007)&	N	-	-	-
Sudan	**1** - (2007–2012)	N	**-**	**1** - (2010)	Y (PE)
South Sudan	**7** - (2006–2011)	Y	**-**	**6** - (2009)	N
Swaziland	**3** - (2008–2015)	Y (PE)	**4** - (2011)	**3** - (2008)	Y (PE)
Tanzania	**6** - (2008–2013)&	N	**-**	**10** - (2009)	Y
Togo	**4** - (2011–2015)	N	**0** - (2011)	**0** - (2009)	N
Uganda	**1** - (2011–2015)&	N	**5** - (2011)	**11** - (2010)	N
Zambia	**5** - (2011–2015)&	Y	**10** - (2010)	**7** - (2007)	N
Zanzibar	**3** - (2007–2012)	N	**3** - (2011)	**5** - (2008)	N
Zimbabwe	**1** - (2008–2013)	N	**4** - (2011)	**1** - (2010)	Y

# See Table 1 for definitions of map codes (1–11);

* E: NMS goal is elimination;

** PE: NMS aims to achieve pre-elimination;

& M&E plan available; Cells shaded in grey indicate that no report is available.

Twenty (42.5%) national strategies gave evidence of sub-national planning whether or not this evidence was linked to the risk stratification map used within the NMS. It is notable that 18 of these NMS gave clear evidence of planning control activities and targeting resources at sub-national level. However, neither Cape Verde nor the Comoros present a risk map although both countries are aiming to achieve elimination and pre-elimination conditions respectively and detailed case reconnaissance is a pre-requisite of the attack phase of an elimination strategy.

### Malaria Programme Performance Reviews

All MPRs except Burundi (2011) and Togo (2011) presented a malaria risk map. Five of the MPRs (Comoros, Djibouti, Ethiopia, Madagascar and Mauritania) have used either a stratification based either on a qualitative epidemiological description or on eco-climatic factors (map codes 1 and 2; Table 2); 8 MPRs (Botswana, Mozambique, Rwanda, Senegal, Swaziland, Uganda, Zanzibar and Zimbabwe) used maps based on HMIS or national prevalence survey data (map codes 3–5; Table 2); and 4 countries (Kenya, Malawi, Namibia and Zambia) used modeled malaria parasite prevalence maps developed using country level data (map code 10; Table 2). Reviewing the narratives and other information linked to the maps provided in the MPRs, only 5 (Botswana, Kenya, Swaziland, Zanzibar and Zimbabwe) provided evidence of sub-national level planning or targeting of interventions (Table 2). According to their NMSs and compared with other countries analyzed here, these countries either have ambitious goals for malaria control including elimination of the disease in the near future (Botswana, Swaziland and Zanzibar) or aggressive control programmes (Kenya and Zimbabwe).

None of the MPRs mapped or summarized disease burdens at sub-national level and only a few attempted to describe either the geographical distribution of disease burden or the seasonality of transmission as recommended by WHO-AFRO. Seasonality of malaria transmission was defined in only two MPRs (Rwanda and Swaziland). It is notable that countries like Botswana, where malaria is markedly seasonal [37] did not include a definition of seasonality. Madagascar is the only country that included cartography of malaria parasite species distribution.

### Global Fund applications

From the review of 43 most recent, successful GF applications, 35 (81%) presented a risk map. No risk maps were available in GF applications for Burundi, CAR, Comoros, The Gambia, Madagascar, Malawi, Namibia and Togo (map code 0; Table 2). A wide range of epidemiological stratifications was used based on a rich variety of data sources among the applications that included a map. 10 countries used either a qualitative descriptive epidemiological or eco-climatic stratification (map codes 1 and 2; Table 2); 13 countries used maps based on national case, incidence or prevalence data summarized according to administrative units (map codes 3–5; Table 2); 6 countries used a MARA/ARMA climate based map (map codes 6–8; Table 2) and 6 applications used models of predicted malaria prevalence (map codes 9–11; Table 2). GF applications showed a preference for mapped data from routine or national sample surveys (N = 13; map codes 3–5; Table 2) or epidemiological descriptions based largely on expert opinion and eco-climatic factors (N = 13; map codes 1 and 2; Table 2). Web-available public domain products from either the MARA/ARMA or the MAP websites were used less frequently (N = 6; map codes 6–9; Table 2). The MAP modeled prediction of *Pf*PR_2–10_ (map code 11; Table 2) was presented in the GF Round 10 applications for Uganda and as a second map in the GF application for Sierra Leone.

Fifteen (35%) of the 43 applications reviewed had evidence of sub-national design of control. Only 3 of these (Comoros, CAR and Madagascar) did not include a risk map (Table 2). Kenya, Somalia, Swaziland and Zimbabwe represent the most notable, strategic uses of maps to define intervention approaches and priority tailored needs. These countries show evidence of linking the choices for malaria control intervention directly to the epidemiological stratification in making their business case to the GF. For example, Kenya used a modeled map of predicted *Pf*PR_2–10_ that is based on national prevalence surveys and developed in collaboration with the NMCP. The map shows a range of transmission intensities across the country and epidemiologically tailored packages of interventions are defined for each transmission level. Long Lasting Insecticide-treated Nets (LLINs), Insecticide Residual Spraying (IRS), Artemisinin Combination Therapies/Rapid Diagnostic Tests (ACT/RDT) and IPTp are prioritised at the higher extremes of transmission while RDT, ACT and surveillance are the key interventions in low transmission settings. In Somalia, higher transmission areas are targeted for mass distribution of LLINs and maintenance once coverage reaches >90%. Transmission foci, targeted for annual spray campaigns, exist in areas of low transmission in Puntland and Somaliland. The foci are selected on the basis of a prevalence of <5% and no reported malaria epidemics in the last ten years. Somalia's map is also based on modeled *Pf*PR and developed in collaboration with the NMCP. Zimbabwe has used maps to identify districts targeted for elimination and under preparation for pre-elimination. Interventions in other districts are selected according to the local epidemiology, for example, sustained LLIN use in high transmission districts and specific targeted interventions for mobile populations. Swaziland is also preparing for pre-elimination and the NMCP has set up a rigorous system for rapid identification and mapping of transmission foci at community level through active and passive case detection. In some cases, maps of current coverage of interventions like ITN or IRS are also presented (*e.g.* Malawi, Somalia, Uganda, Zambia, Zanzibar and Zimbabwe) although these are not necessarily tied to epidemiological risk maps.

## Discussion

Although malaria risk maps have been used in Africa for over 80 years, a more recent renaissance in risk mapping has been fuelled by the proliferation of more sophisticated techniques for spatiotemporal analysis available to the health sector. However the control of malaria is still to benefit from these new mapping tools. Although countries are urged to use the epidemiology of malaria risk to frame national strategic plans, select evidence-based intervention packages and rationalize resource requests in applications for domestic support and ODA, there are very few examples where this has been done and even fewer examples of the use of modern techniques in risk mapping within national planning.

**Figure 1:**
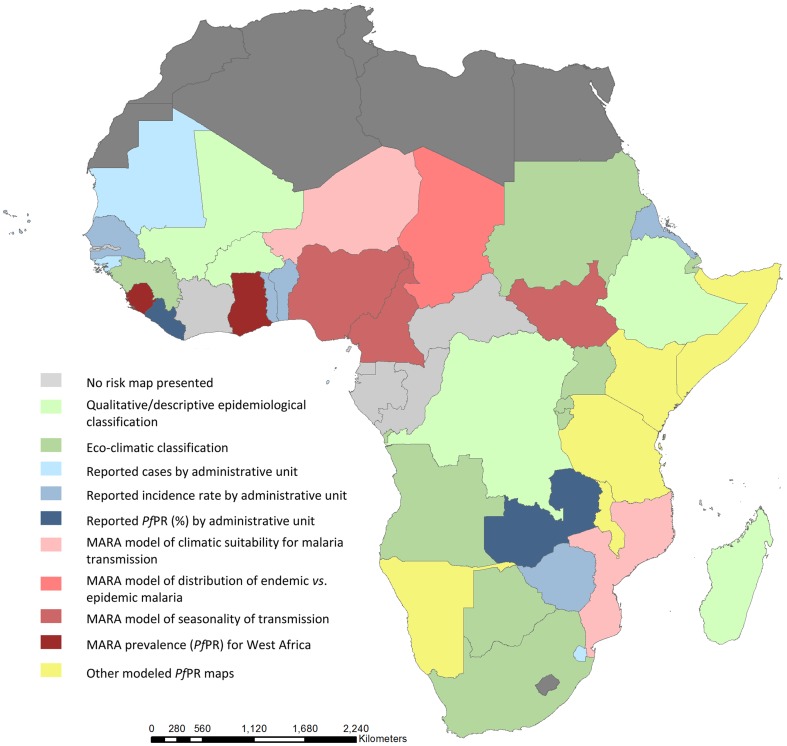
Map showing types of the most recent malaria maps used by NMCPs in Global Fund applications, National Malaria Strategies, and Malaria Programme Reviews.

This review examines strategic, policy and funding request reports for malaria endemic countries in Africa. A malaria risk map is available in at least one of these policy documents for 43 (91%) endemic countries and the most recent map used by each country across all reports reviewed is summarized according to map types in Table 3 and Figure 1. Countries use a variety of maps and the map selected appears to be based on a range of factors. Key among these is the semblance of the map to what is known about the distribution of the disease through expert opinion. This review observed that maps that describe a wider range of transmission across a country are more frequently used than those that crudely group transmission within a few categories. Choice is also dependent on the intended use of the map for example, applications for ODA tend to include maps that show current burdens of disease based on routine HMIS data, national prevalence data or historically established and trusted expert opinion maps (map Codes 1–5).

**Table 3:** Summary of types of maps used in most recent available report by country.

Code	Description of map used	Countries (most recent available report – date)	N^o^ of countries
0	No risk map available in any of the reports reviewed	**Central African Republic** (GF R8), **Equatorial Guinea** (NMS 2009–2013), **Gabon** (NMS 2006–2012), **The** **Gambia** (NMS 2008–2015)	4
1	Qualitative/descriptive epidemiological classification	**Angola** (GF R10), **Botswana** (NMS 2010–2015), **Burundi** (NMS 2008–2012), **Comoros** (MPR 2011), **Djibouti** (MPR 2011), **Guinea** (GF R10), **Sudan** (GF R10).	7
2	Eco-climatic classification	**DRC** (GF R10), **Ethiopia** (MPR 2011), **Madagascar** (MPR 2011), **Mali** (NMS 2010–2014), **Mauritania** (MPR 2011)	5
3	Reported cases by administrative unit	**Republic of Congo** (GF R8), **Guinea Bissau** (GF R9), **São Tomé & Príncipe** (GF R7), **Zanzibar** (MPR 2011)	4
4	Reported incidence by administrative unit	**Benin** (GF R7), **Burkina Faso** (NMS 2011–2015), **Cape Verde** (GF R10), **Côte d'Ivoire** (GF R8), **Eritrea** (GF R9), **Mozambique** (NMS 2011–2016), **Senegal** (NMS 2011–2015), **South Africa** (MoH Ann. Rep. 2007), **Swaziland** (MPR 2011), **Togo** (NMS 2011–2015), **Zimbabwe** (MPR 2011)	11
5	Reported *Pf*PR by region	**Liberia** (GF R10), **Rwanda** (MPR 2011), **Uganda** (MPR 2011)	3
6	MARA/ARMA model of climatic suitability for transmission	**South Sudan** (GF R9)	1
7	MARA/ARMA model of distribution of endemic *vs*. epidemic malaria	**Chad** (GF R9), **Niger** (GF R7)	2
8	MARA/ARMA model of seasonality of transmission	**Cameroon** (GF R9), **Nigeria** (NMS 2009–2013)	2
9	MARA/ARMA prevalence (*Pf*PR) model for West Africa	**Ghana** (NMS 2008–2015), **Sierra Leone** (GF R10)	2
10	Other country-level modeled *Pf*PR maps	**Kenya** (GF R10), **Malawi** (NMS 2011–2015), **Namibia** (NMS 2010–2016), **Somalia** (NMS 2011–2015), **Tanzania** (GF R9), **Zambia** (NMS 2011–2015).	6
11	MAP products		0

An additional observation is that maps vary widely in the types of information they portray. This may reflect the accessibility of existing maps to national control programmes or may demonstrate that individual countries have different priorities for epidemiological data according to their programme objectives. For example models of predicted *Pf*PR have been used in Malawi, Namibia, Kenya, Zambia and Somalia where regional research partners have worked with NMCPs. Countries in more arid regions where rainfall is well recognised as a key driver of transmission have shown a preference for the range of climate-based MARA/ARMA models (*e.g.* South Sudan, Chad, Niger, Cameroon and Nigeria). Additionally MARA/ARMA adopted a successful model to have a regional base and focus and participation of control stakeholders across Africa. MARA/ARMA maps still adorn many national malaria control programme office walls today

National programmes increasingly portray maps of case incidence summarized by administrative unit; 11 (25%) of the most recent maps used across all policy documents were incidence maps (Table 3; map code 4). It is likely that the trend towards using incidence will increase as control targets are set towards achieving zero cases, zero deaths, specified disease burden reductions or elimination as their strategic goal. This will necessitate a marked improvement in the quality, fidelity and analytical approaches to these data derived from imperfect HIS reporting units. Accompanying footnotes or other information that would allow for the interpretation of actual coverage of HIS data in space and time are rarely provided in the mapped incidence data. Incidence maps are not standardised between countries and in general difficult to analyse against international goals, for example that proposed by WHO to define pre-elimination stages (<1 case per 1000 population per year). There are indications that adapted Geographic Information Systems (GIS) modelling to HIS reported malaria case data is beginning to be explored in Rwanda and Tanzania. In both countries attempts have been made to generate facility-catchment areas and interpolated maps of case prevalence have been developed (Rwanda-MPR 2011) or maps of proportions of outpatient attendance that are due to malaria (Tanzania- GF Round 9).

Other countries have expressly defined spatial ambitions, for example, to achieve five malaria-free districts (Zambia) or to maintain malaria-free districts (Namibia) as part of their NMS. Both countries recognize the importance of changing their routine HIS to become more aggressive and will also incorporate routine active case detection. These approaches will place demands on having high spatial resolution health facility and population distribution mapping linked within a GIS in order to identify residual foci of malaria transmission. New techniques to describe and enumerate facility catchment populations [38] and enhance the value of incomplete HIS data [39] are under development in the field of health model-based geostatistics and malaria case incidence mapping could benefit from this science.

The renewed interest in malaria risk mapping coincides with a period of massively increased computational power to run complex models, thus enabling a better handling of sparse, overly-distributed malaria data and other epidemiological information. This capacity to map malaria data was not available to malariologists 50 years ago. However the computational and skill requirements of model-based geo-statistics often restricts new tools for malaria cartography to the hands of scientists located in parts of the world with ready access to stable, large-scale computing capacity. This disconnect between malaria control specialists and epidemiologists working in endemic areas of Africa and those driving the science and products of malaria mapping, might explain why new web-enabled products are used infrequently in the countries that need them most.

As far as could be defined within the national strategies, programme reviews or ODA requests only a few countries tied the geographical variation of malaria risk to specifically tailored sub-national intervention plans or resource allocation. Notable among these were Zambia, Zimbabwe, Somalia and Kenya. These countries all have access to risk data assembled at sub-national decision-making units, an important pre-requisite for summarizing risk. Spatially continuous maps of predicted risk are likely to be less valuable for planning than risk summarised according to geographical decision-making units. Available risk map products are not linked to empirical models that predict the outcome of investment in single *versus* combined interventions provided at varying levels of coverage. There has, however, been a growth in mathematical modelling of predicted intervention impact based on starting malaria risks [40]–[42] and a renewed interest in defining the continuum of malaria endemicity to guide countries in their decisions about malaria elimination that use combinations of current and historical parasite prevalence (*Pf*PR_2–10_<1%) and case-incidence (<1 case per 1000 population) within defined geographical areas [43]–[46]. These epidemiological planning tools have yet to be effectively linked to the science of malaria risk mapping and subsequently used effectively with risk maps to guide sub-national policy.

## Conclusion

The successes of recent investment in malaria control will be lost unless a more rational basis for financing is established. Investing in high burden, poorly served, densely populated areas of Africa, or within a single country, will focus limited resources where they are most needed to sustain reductions in disease burden. Re-defining packages of interventions across a spectrum of malaria risk will provide a more intelligent basis to reach different measurable milestones with time. These may seem obvious, but to-date, the effective assembly of data from multiple sources and integrating these in novel ways to define risk, intervention, financial needs and priorities for future monitoring has been poor and there is a need to explore ways to improve how data are used, managed and brokered for more effective malaria control within the region. As countries invest in the collection of point prevalence malariometric data and higher quality HMIS data, it is important to develop the science behind interpolating and interpreting these data. Ensuring country ownership of epidemiological risk maps and research outputs will enhance their long-term value and application. With time, international donor agencies and regional support networks will need to evaluate whether epidemiologically stratified sub-national policies are applied in practice. This will require both an analysis of district-level business plans and data on the coverage of interventions at sub-national level.
